# Scoping review of risk factors of and interventions for adolescent repeat pregnancies: A public health perspective

**DOI:** 10.4102/phcfm.v10i1.1685

**Published:** 2018-06-19

**Authors:** Desiree Govender, Saloshni Naidoo, Myra Taylor

**Affiliations:** 1 KwaZulu-Natal Department of Health, University of KwaZulu-Natal, South Africa; 2 Discipline of Public Health, University of KwaZulu-Natal, South Africa

## Abstract

**Background:**

Adolescent repeat pregnancy is of importance in public health because the birth of a second child to an adolescent mother compounds the adverse medical, educational, socio-economic and parenting outcomes. Repeat pregnancy in adolescence is not only an international phenomenon but also a local concern as it also occurs in South Africa. The prevalence of adolescent repeat pregnancy in Durban, KwaZulu-Natal, was reported as 17.6% in 2013.

**Aim:**

This review aimed to gather relevant information from national and international sources to inform practice and to provide an understanding of what is known about the risk factors of and the interventions for adolescent repeat pregnancy.

**Methods:**

A scoping review was undertaken using the Arksey and O’Malley framework. An electronic search was conducted using PubMed, Medline, Science Direct, Ebscohost, Sage and Wiley Online and Google Scholar.

**Results:**

The search identified 3032 citations. After a review of the full text articles, 26 articles met the inclusion criteria. Risk factors pertaining to adolescent repeat pregnancy are categorised according to individual factors, partner relationship factors, family factors, peer factors, and social and community factors. Interventions to reduce adolescent repeat pregnancy have been largely influenced by the ecological framework. Across studies, adolescent mothers who received medical, psychosocial, educational, and family planning support experienced lower rates of repeat pregnancy.

**Conclusion:**

A single ‘one-size-fits-all’ intervention for adolescent repeat pregnancy prevention is unlikely as different strategies were employed by the intervention programmes in this scoping review.

## Introduction

Adolescent childbearing and repeat pregnancy are both public health and social problems.^1^ Darroch et al.^2^ reported that worldwide approximately 21 million girls in the 15–19 years age category became pregnant in 2016.^2^ Furthermore, adolescent pregnancy and childbirth complications are a leading cause of the global burden of poor maternal health conditions and death in the 15–19 years age category.^3^ The prevalence of adolescent repeat pregnancy is alarming. Approximately 12% – 49% of adolescent repeat pregnancies in the United States of America (USA) occur within 1 year of the previous pregnancy.^4^ Supporting data from Australia and Canada indicate that the prevalence of adolescent repeat pregnancy in these countries is 33% and 15.2%, respectively.^5,6^ In the United Kingdom, one-fifth of births to adolescents under 18 years of age are repeat pregnancies.^7^ Repeat pregnancy amongst adolescents is not only an international phenomenon, but it also occurs in South Africa. Though the national prevalence of adolescent repeat pregnancy is not known in South Africa, the prevalence in Durban, KwaZulu-Natal was reported to be 17.6% in 2013.^8^

Adolescent repeat pregnancy is of particular importance in public health because the birth of a second child to an adolescent mother compounds the adverse medical, educational, socio-economic and parenting outcomes.^9,10^ In addition, girls who have repeat adolescent pregnancies generally experience suicidal ideation, depression and anxiety.^11^Adolescent parenting stress also increases with rapid repeat pregnancy, which could result in neglect of the second child and negative parenting behaviour.^12^ Adolescent parents bearing two or more children within a period of 5 years are more likely to rely on social grants, drop out of school and experience additional childbearing within shorter birth intervals.^4,10,13^ In the USA, taxpayers contribute almost $7 billion towards the burden of adolescent childbearing which includes health care, criminal justice, foster care, and public social and economic assistance.^13^

Researchers have often referred to the topic of adolescent childbearing and rapid repeat pregnancy as a phenomenon or an enigma.^7,12,14^ A range of opinions have been forwarded amongst public health professionals, clinicians, social scientists, advocacy groups and the media regarding the issue of adolescent pregnancy. The current debate centres on the conceptualisation of adolescent pregnancy as a public health problem versus adolescent pregnancy as a reproductive choice and process. Adolescent pregnancy is culturally accepted in different parts of the world despite being labelled a public health problem. Some adolescent health experts have refuted the claim that adolescent pregnancy results in catastrophe for the mother and her infant.^15,16^ Macleod^15^ argues that the moral judgment of adolescent pregnancy has now been substituted by ‘scientific scrutiny’ (p. 59).

A study by Smith and Pell^17^ found a causal association between repeat adolescent pregnancy and poor birth outcomes. However, these findings were dismissed on the stance that adolescent repeat pregnancy is not a public health problem and that birth outcomes are largely influenced by confounders such as socio-economic circumstances.^18^ Criticising Lawlor and Shaw’s^18^ argument that adolescent pregnancy is not a public health problem, Scally^19^ argues that adolescent pregnancy requires integrated public health action through several sectors to help adolescents prevent unwanted pregnancies and manage the health, economic and social consequences of the pregnancy and birth. In support of Scally’s^19^ assertions, Rich-Edwards^20^ advances the explanation that poverty is a risk factor for adolescent pregnancy and that premature parenthood influences the cycle of future poverty, making this a public health problem.

On the basis of these arguments, the literature review was essential in facilitating a better understanding of the public health approach towards adolescent repeat pregnancy. Understanding the risk factors of and the interventions for adolescent repeat pregnancy can provide guidance to health practitioners and decision makers. This scoping review was designed with the purpose of gathering relevant information from national and international sources to inform practice and to provide an understanding of what is known about the risk factors of and the interventions for adolescent repeat pregnancy.

## Defining repeat adolescent pregnancy

The literature distinguishes between adolescent repeat pregnancy and rapid adolescent repeat pregnancy. Adolescent repeat pregnancy is defined as a second pregnancy or additional pregnancies to a woman younger than 20 years of age.^6^ Most research to date has focused on rapid repeat pregnancy amongst adolescents.^21,22,23,24,25,26^ Rapid adolescent repeat pregnancy is defined as a second birth or pregnancy that occurs within 2 years of the previous pregnancy.^24^

## Methods

This literature review employed a scoping review methodology based on the framework by Arksey and O’Malley^27^ and the recommendations put forward by Levac et al.^28^ According to Davies et al.,^29^ ‘… scoping involves the synthesis and analysis of a wide range of research and non-research material to provide greater conceptual clarity about a specific topic or field of evidence’ (p. 1386). The study followed the stages of the Arksey and O’Malley^27^ framework which included: (1) identifying the research question, (2) identifying relevant studies, (3) study selection, (4) charting the data and (5) collating, summarising and reporting the results.

### The research question

The research question was generated by our public health concerns about adolescent repeat pregnancy. The scoping review was guided by the research question: ‘what are the risk factors of, and the interventions for adolescent repeat pregnancy?’ A key aspect of a scoping review is a broad and comprehensive research question to provide breadth of the literature.

### The data sources and search strategy

The databases used to conduct the literature search were selected on the basis that the topic of adolescent repeat pregnancy encompasses social science research and public health research. The databases included PubMed, Medline, Science Direct, Ebscohost and Wiley Online. Search engines included Google and Google Scholar. The search terms included ‘adolescent pregnancy’ and/or ‘adolescent repeat pregnancy’ and/or ‘adolescent parenting’ and/or ‘adolescent repeat pregnancy risk factors’ and/or ‘consequences of adolescent pregnancy’ and/or ‘secondary pregnancy prevention’. The snowballing technique was applied by identifying references in the scrutinised review articles to obtain detailed and relevant information. Searching involved published literature from 1990 to 2016.

### Study selection

**FIGURE 1:**
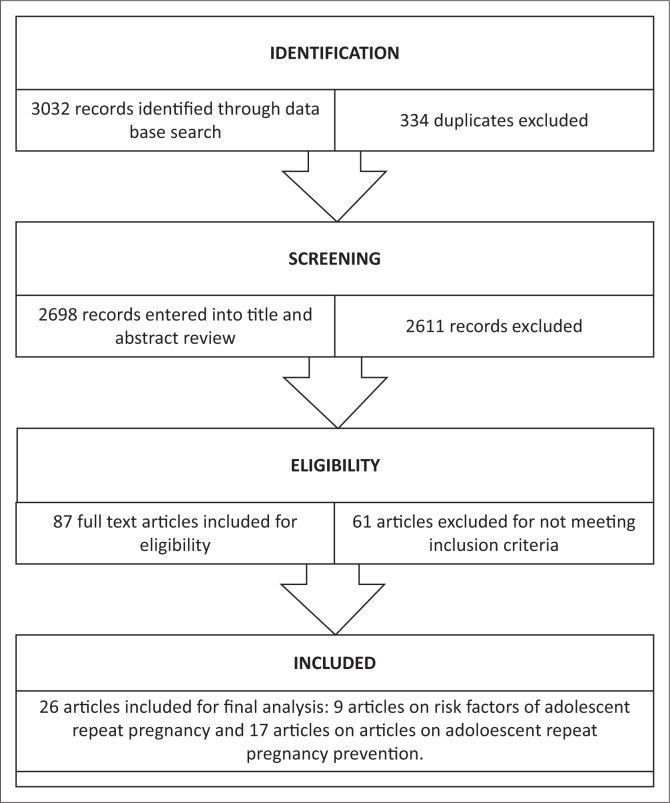
Article selection process.

When the relevant literature was identified, the inclusion and exclusion criteria were established (Table 1). The papers that were included for this scoping study comprised published peer review studies, review articles and opinion articles. Figure 1; depicts the scoping process which includes the number of publications retrieved and selected from the database search.

**TABLE 1:** Inclusion and exclusion criteria.

Inclusion	Exclusion
• Articles written in English.	• Articles not written in English.
• Articles involving a secondary or repeat pregnancy prevention intervention for adolescents.	• Articles focusing on primary pregnancy prevention for adolescents.
• Study participants were defined as adolescents (13 to 19 years of age).	• Study participants who were not defined as adolescents.
• Articles identifying risk factors of repeat adolescent pregnancy.	• Articles published before 1990.
• Intervention studies with baseline and post-intervention data.	
• The outcome of interest had to include reduced adolescent repeat pregnancy rate.	

### Charting the data

An electronic data spreadsheet was developed. Data were extracted according to the following details: author information, title, journal, year of publication, identified risk factors, study design, study setting, participants, intervention and the related health outcome.

## Ethical Considerations

This literature review was part of a larger doctoral study that had been approved by the University of KwaZulu-Natal Bioethics Research Committee (ref no: BFC553/16) and the KwaZulu-Natal Department of Health (ref no. KZ_2016RP26_545).

## Results

### Description of studies

The online searches yielded 3032 relevant citations of which 2698 were eligible for title and abstract review. Of these, a total of 87 articles were retrieved for full text screening. After review of the full text articles, 26 articles met the inclusion criteria (illustrated in Figure 1). The articles were summarised into themes of risk factors and of interventions for adolescent repeat pregnancy. Nine of the 26 articles addressed the risk factors of adolescent repeat pregnancy. The majority of the studies (67%) addressing the risk factors of adolescent repeat pregnancy were from the USA. The description of included studies for the risk factors of adolescent repeat pregnancy is presented in Table 2. Seventeen articles in this scoping review addressed interventions on adolescent repeat pregnancy. Details of these 17 articles included for the analysis and discussion of the interventions on adolescent repeat pregnancy are presented in Table 3.

**TABLE 2:** Description of included studies for risk factors of adolescent repeat pregnancy.

Author (year)	Country	Title	Study design	Number of participants
Boardman et al. (2006)	USA	Risk factors for unintended versus intended rapid repeat pregnancies amongst adolescents.	Retrospective observational cohort	1117
Crittenden et al. (2009)	USA	The role of mental health factors, behavioral factors and past experiences in the prediction of rapid repeat pregnancy in adolescents.	Retrospective cohort study	357
Jacoby et al. (1999)	USA	Rapid repeat pregnancy and experiences of interpersonal violence amongst low-income individuals.	Case control study using retrospective chart review	100
Lewis et al. (2010)	Australia	Predictors of sexual intercourse and rapid repeat pregnancy amongst teenage mothers: An Australian prospective longitudinal study.	Prospective longitudinal study	147
Mphatswe et al. (2016)	South Africa	Prevalence of repeat pregnancies and associated factors amongst teenage mothers in KwaZulu-Natal.	Prospective observational study	341
Pfitzner et al. (2003)	USA	Predictors of repeat pregnancy in program for pregnant teens.	Retrospective case control study	1838
Raneri and Wiemann (2007)	USA	Social ecological predictors of adolescent repeat pregnancy.	Retrospective cohort study	932
Rowland (2010)	UK	Social predictors of repeat adolescent pregnancy and focused strategies.	Literature review	N/A
Rigsby et al. (1998)	USA	Risk factors for rapid repeat pregnancy amongst adolescent mothers: A review of the literature.	Systematic review	Included 20 studies for analysis

Note: Please see the full reference list of the article, Govender D, Naidoo S, Taylor M. Scoping review of risk factors of and interventions for adolescent repeat pregnancy: A public health perspective. Afr J Prm Health Care Fam Med. 2018;10(1), a1685. https://doi.org/10.4102/phcfm.v10i1.1685, for more information.

USA, United states; UK, United Kingdom; N/A, not applicable.

**TABLE 3:** Description of the intervention studies on adolescent repeat pregnancy.

Author (year)	Country	Title	Study design	Number of participants	Intervention
Barnet et al. (2007)	USA	Home visiting for adolescent mothers; effects of parenting, maternal life course and primary care linkage.	Randomised control trial (RCT)	*N* = 84 Intervention = 44 Control = 40	Community-based home visiting focusing on parenting education, sexual education, completion of school and communication.
Barnet et al. (2009)		Motivational intervention to reduce rapid subsequent births to adolescent mothers.	Randomised control trial	*N* = 235 Three groups: Intervention 1: (CAMI +) = 80 Intervention 2: (CAMI only) = 87 Control (usual care) = 66	Computer-assisted motivational intervention (CAMI). Trained CAMI counsellors provided motivation on use of contraception and condoms. The CAMI + group received education on infant development and care, feeding and nutrition, sexual and reproductive health, educational attainment and goal setting via biweekly home visits. The CAMI only group were visited at home on a quarterly basis. Duration of intervention was 24 months.
Black et al. (2006)	USA	Delayed second births by paraprofessionals and nurses: A randomised control trial of a home-based mentoring programme.	Randomised control trial	*N* = 181 Intervention = 87 Control = 94	Home-based mentoring programme (‘Big sister approach’). Home visits which included education on contraceptive education, infant care, behaviour skills and communication skills. Duration of intervention was 24 months.
Cohen et al. (2016)	USA	Twelve-month contraceptive continuation and repeat pregnancy amongst young mothers choosing post-delivery contraceptive implants or post- placental intrauterine device.	Prospective cohort	*N* = 244 Intrauterine device (IUD) = 82 Contraceptive implants = 162	Of the 82 young mothers choosing the IUD, 74 used the levonorgestrel whilst 8 used the copper IUD. One hundred and 62 chose the etonogestrel implant. The follow-up period was 12 months.
Corocan and Pillai (2007)	USA	Effectiveness of secondary pregnancy prevention programs: A meta-analysis.	Meta-analysis	Included 16 studies for analysis	N/A
Cox et al. (2012)	USA	Evaluation of raising adolescent families together program: a medical home for adolescent mothers and their children.	Prospective single cohort	*N* = 181	The medical home intervention consisted of infant care education, life orientation, social services support, family support groups, contraceptive education, day care and community outreach services. Duration of intervention was 24 months.
Damle et al. (2015)	USA	Early initiation of postpartum contraception: Does it decrease rapid repeat pregnancy in adolescents?	Retrospective chart review	*N* = 340	Intervention included the provision of long-acting reversible contraception (intrauterine device or etonogestrel implant). Duration of the follow-up was 24 months.
Drayton et al. (2000)	Jamaica	The impact of the Women’s Centre of Jamaica Foundation Programme for adolescent mothers on repeat pregnancies.	Retrospective cohort	*N* = 266 Intervention = 87 Comparison = 173	The Women’s Centre of Jamaica Foundation Programme provided community outreach services, infant care education, family planning services, life skills orientation, educational attainment support and job skills. Repeat pregnancies were measured during 1995–1998.
Ford et al. (2002)	USA	Effects of a prenatal care intervention for adolescent mothers on birth weight, repeat pregnancy and educational outcomes at one year.	Randomised control trial	*N* = 282 Intervention = 165 Control = 117	In the prenatal care intervention, adolescents were paired in groups. The adolescent mothers received infant care education, labour preparation, nutrition education and postpartum care.
Key et al. (2008)	USA	Effectiveness of an intensive, school-based intervention for teen mothers.	Prospective cohort	*N* = 315 Intervention = 63 Comparison group = 252	School-based intervention that included social services, medical care, peer-based education, contraceptive education, parenting skills and educational attainment support. Follow-up was done at age 20 or 3 years after the index birth.
Lewis et al. (2000)	Australia	Implanon as a contraceptive choice for teenage mothers: a comparison of contraceptive choices, acceptability and repeat pregnancy.	Prospective cohort	*N* = 189 Implanon =73 Combined oral contraceptive pill (COCP) or depot medroxyprogesterone acetate (DMPA) = 40 Barrier method or none = 24	The intervention included the provision of contraception. The duration of the follow-up was 24 months.
O’Sullivan and Jacobsen (1992)	USA	A randomised control trial of a health care program for first time adolescent mothers and their infants.	Randomised control trial	*N* = 243 Intervention = 132 Control = 132	Intervention included behaviour skills development, contraceptive education, life option enhancement, sexuality, HIV, and sexually transmitted infections (STI) education, infant care and education support attainment. The duration of the intervention was 18 months.
Rabin and Seltzer (1991)	USA	The long term benefits of a comprehensive teenage pregnancy program.	Quasi-experimental	*N* = 589 Intervention = 498 Comparison = 91	The Queens Hospital Centre Teenage Programme included components such as abstinence, community outreach services, contraceptive education, life option enhancement, sexuality, HIV and STI education and educational attainment support. The study followed the mothers until they reached 20 years.
Seitz and Apfel (1993)	USA	Adolescent mothers and repeated child bearing: Effects of a school-based intervention program.	Quasi-experimental	*N* = 100 Intervention (seven week) = 50 Intervention (more than 7 weeks) = 52	The intervention at the Polly T McCabe Centre included family planning, child bearing, infant care and educational attainment support. Follow-up was done at 24 months and 60 months.
Solomon and Leifeld (1998)	USA	Effectiveness of a family support centre approach to adolescent mothers: Repeat pregnancy and school dropout.	Randomised control trial	*N* = 88 Intervention = 88 Control = 39	The Family Growth Centre (FGC) provided home visits, health education, infant care, contraception education and access, life skills orientation, community outreach services, sexuality, HIV and STI education and behaviour skills development. Follow-up was done at 24 months.
Steven Simon et al. (1999)	USA	Preventing repeat adolescent pregnancies with early adoption of the contraceptive implant.	Prospective observational study	*N* = 354 Intervention = 171 Control = 138	Intervention group chose implant as their contraceptive method Follow-up was done at 24 months.
Tocce et al. (2012)	USA	Rapid repeat pregnancies in adolescents: Do immediate postpartum contraceptive implants make a difference.	Prospective observational study	*N* = 396 Intervention = 171 Control = 225	The intervention group received immediate postpartum etonogestrel implants. Follow-up was done at 12 months.

Note: Please see the full reference list of the article, Govender D, Naidoo S, Taylor M. Scoping review of risk factors of and interventions for adolescent repeat pregnancy: A public health perspective. Afr J Prm Health Care Fam Med. 2018;10(1), a1685. https://doi.org/10.4102/phcfm.v10i1.1685, for more information.

USA, United states; UK, United Kingdom; N/A, not applicable.

Rowland^30^ demonstrates a comprehensive ecological model for the predictors of rapid repeat pregnancy amongst adolescent mothers. For adolescents experiencing a repeat pregnancy, the risk factors are categorised according to individual factors, partner relationship factors, family factors, peer and school factors and social and community factors (Table 4).

**TABLE 4:** Risk factors for adolescent repeat pregnancy.

Individual factors	Family factors	Peer and school factors	Partner relationship factors	Social and community factors
Race	Having a mother with low educational status.	Dropping out of school after first pregnancy.	Having an older partner (three or more years older).	Lower socio-economic status.
Ethnicity	Intergenerational adolescent pregnancy.	Adolescent mother fails to return to school six months postpartum.	Intimate partner violence.	Low education levels.
Depression and suicidal ideation	Lack of family support.	Having more friends who are adolescent mothers or have experienced adolescent pregnancy.	Failed relationship with the partner responsible for index pregnancy.	Cultural and societal norms regarding childbearing and parenting.
Religiosity	Dysfunctional family-poor mother–daughter relationship.	Low educational motivation.	In relationship with new partner postpartum.	-
Risky sexual behaviour or recommencement of sexual intercourse less than six months postpartum or ongoing sexual intercourse for more than three months post-delivery.	Abuse and neglect.	Friends place emphasis on childbearing and motherhood.	Married at a young age (adolescent).	-
Having more than one sexual partner in the last 12 months postpartum.	Domestic violence.	-	Partner pregnancy intention (wants a repeat pregnancy).	-
Aggression	Family places emphasis on childbearing and motherhood.	-	-	-
Age of first pregnancy.	Difficult relationship with family.	-	-	-
Contraception preferences (oral contraception versus long-acting contraceptive).	-	-	-	-
Planned first pregnancy.	-	-	-	-
Positive attitudes towards childbearing and parenting.	-	-	-	-
Cognitive function	-	-	-	-
Adolescent mother living with her mother after delivery of first child. Child reared by grandmother who provides socio-economic support.	-	-	-	-
Poor obstetrical outcome such as miscarriage.	-	-	-	-

The individual factors relating to adolescent repeat pregnancy include race, ethnicity, age of first pregnancy, int. ended first pregnancy and attitudes towards early childbearing^10,30,31^ In addition, cognitive functioning and a history of depression have been linked to adolescent repeat pregnancies.^14^ Lewis et al.^22^ found that repeat pregnancy was more likely in indigenous Australian adolescents (odds ratio 2.38, 95% confidence interval (CI) 1.38–4.11). Poor obstetrical outcomes such as miscarriages have been associated with rapid repeat adolescent pregnancy. Partner relationship factors such as living with an older partner, being married at a young age, having a new partner and intimate partner violence are more common in those adolescents with a repeat pregnancy.^30,31^ Family factors that are related to subsequent adolescent repeat pregnancies include dysfunctional mother-daughter relationships, having mothers with low educational levels, intergenerational adolescent pregnancy, and lack of family support.^30,31^

Equally important, an adolescent mother who lives with her mother after the birth of her first child and relies on her mother for financial and social support is more likely to experience a repeat adolescent pregnancy.^30,31^ Peer factors in particular, such as association with friends who are adolescent parents, postpartum school enrolment and low educational ambition, have been linked with adolescent repeat pregnancy.^30,32^ The social and community factors associated with adolescent repeat pregnancy include low socio-economic status, low educational status, and society norms that accept adolescent childbearing.^30,31,32^

These ecological factors have been tested in studies conducted by Lewis et al.,^22^ Jacoby et al.,^23^ and Raneri and Wiemann.^24^ Jacoby et al.^23^ found a strong association between adolescent rapid repeat pregnancy and interpersonal violence (*p* < 0.00001; odds ratio 22.6). Similarly, Raneri and Wiemann^24^ demonstrated that adolescents with repeat pregnancies had been physically abused by their boyfriends or husbands within the first 3 months of the delivery (odds ratio 1.85, 95% CI: 1.18–2.88). Lewis et al.^22^ acknowledged that the following factors increased the risk of adolescent repeat pregnancy amongst Australian adolescent mothers: (1) living with the father of the child (*p* < 0.001), (2) recommencement of sexual intercourse before 6 weeks postpartum (*p* < 0.005), (3) oral contraceptive use (*p* < 0.005) and (4) ongoing sexual intercourse > 3 months (*p* < 0.005).

Raneri and Wiemann et al.^24^ also recognised that the risk of adolescent repeat pregnancy correlated positively with the following: (1) plans to have a baby in ≤ 5 years (odds ratio 1.55, 1.03–2.34), (2) not using a long-acting contraceptive within 3 months after delivery (odds ratio 2.38, 95% CI: 1.61–3.52), (3) not in a relationship with the father of the first child 3 months after delivery (odds ratio 2.04, 95% CI: 1.37–3.05), (4) father of first child > 3 years older (odds ratio 1.60, 95% CI: 1.10–2.35), (5) not enrolled in school 3 months after delivery (odds ratio 1.75, 95% CI: 1.20–2.55) and (6) ≥ half of friends were adolescent mothers at delivery (odds ratio 1.52, 95% CI: 1.03–2.26).

In the South African context, Mphatswe et al.^8^ found that adolescents with repeat pregnancies were involved with partners who were 5 years or older than them, or they had had multiple sexual partners in the previous 12 months. The prevalence of HIV was also higher in South African adolescent repeat pregnancies than in first time adolescent pregnancies.

According to Raneri and Wiemann^24^ the strongest predictor of adolescent repeat pregnancy is not using a long-acting contraceptive postpartum. Steven-Simons et al.^26^ investigated the adoption of the contraceptive implant to avoid adolescent repeat pregnancy. The authors found that the rate of repeat adolescent pregnancy was significantly lower for early implant users (12%, *p* < 0.0001) than for other adolescent mothers using alternative methods of contraception (46%). Lewis et al.^22^ demonstrated that this claim was warranted and advocated for the provision of long-acting contraceptives to adolescent mothers.

### Interventions to prevent adolescent repeat pregnancy

Interventions to reduce adolescent repeat pregnancy have been largely influenced by the ecological framework of individual, partner, familial, peer, and community factors (Table 5). First time adolescent mothers who attended the Queens Hospital community-based programme in the USA, which provided medical, psychosocial, educational and family planning support through a multidisciplinary team (i.e. a team including a gynaecologist, paediatrician, social worker and health educator), had a lower repeat pregnancy rate (9%) than the control group (70%).^33^

**TABLE 5:** Intervention outcomes.

Author (year)	Adolescent repeat pregnancy	Contraception uptake	Educational attainment or school enrolment
Barnet et al. (2007)	No significant impact on repeat pregnancy. Repeat pregnancy in the intervention group was 45% (14/31) at 24 months whilst control group had a repeat pregnancy rate of 38% (12/32).	-	School enrolment was higher in home visited adolescent mothers (intervention) than in control group (71% vs. 44%, *p* ≤ 0.05).
Barnet et al. (2009)	Lower repeat pregnancy rate in CAMI+ group (13.8%). CAMI only group repeat pregnancy rate was 17.2%. The usual care group (control) had a repeat pregnancy rate of 25.0%.	-	-
Black et al. (2006)	Intention to treat analysis revealed that adolescent mothers in the control group were 2.5 times more likely to have a repeat pregnancy (24% vs. 11%, *p* = 0.05).	-	No differences were noted with regards to maternal educational attainment.
Cohen et al. (2016)	At 12 months follow-up, the repeat pregnancy rate in the adolescent mothers using intrauterine devices was 7.6% (5/67) whilst the repeat pregnancy rate in implant users was 1.5% (2/135).	-	-
Corocan and Pillai (2007)	According to the meta-analysis, secondary repeat pregnancy interventions in the first 19.13 months yielded a 50% reduction in the odds of pregnancy in comparison to the usual care or control groups.	-	-
Cox et al. (2012)	The cumulative repeat pregnancy rate at 12 and 24 months was 14.7% and 24.6%, respectively. The repeat pregnancy rate at 24 months was lower in comparison to benchmark data.	-	-
Damle et al. (2015)	At 8 weeks postpartum, the initiation of LARC decreased the odds of rapid repeat pregnancy within 24 months (OR = 0.018, 95% CI: 0.035–0.307). The repeat pregnancy rate at 24 months was 35%.	-	-
Drayton et al. (2000)	The rate of repeat pregnancy rate was 37% (32/87) amongst WCJF participants versus 60% (104/173) of non-participants. The participants in the WCJF reduced their risk of one or more repeat pregnancies by 45%.	The contraception use was higher amongst WCJF participants (80 of 87) in comparison to 147 of 173 non-participants (*p* = 0.04).	A higher percentage of mothers in the WCJF programme graduated from high school in comparison to non-participants (35% 28/87 vs. 20%, 35/173, *p* = 0.05).
Ford et al. (2002)	At 12 months, the repeat pregnancy rate in the experimental group was 13.4% versus 15.9% in the control group.	-	There were no differences in maternal educational outcomes.
Key et al. (2008)	The rate of repeat pregnancy amongst participants in the school-based intervention was 17% versus 33% in the control group (*p* = 0.001).	-	There were no differences in maternal educational outcomes.
Lewis et al. (2010)	At 24 months, 35% of adolescents had a repeat pregnancy. Adolescents who chose Implanon experienced a repeat pregnancy at 23.8 months (95% CI: 22.2–25.5) whilst adolescents who chose COCP and/or DMPA experienced a repeat pregnancy rate at 18.1 months. Implanon users were less likely to experience subsequent pregnancies.	Adolescents who chose Implanon were more likely to continue the usage of this contraceptive method at 24 months in comparison to those using COCP and/or DMPA (*p* = 0.001).	-
O’Sullivan and Jacobsen (1992)	At 18 months, the repeat pregnancy rate was 12% (13/108) in the intervention group versus 28% (32/118) in the control group. Adolescents in the health care programme were less likely to experience a repeat pregnancy.	-	There were no differences in maternal educational attainment.
Rabin and Seltzer (1991)	There was a 9% repeat pregnancy rate in the intervention group versus 70% in the comparison group.	Eighty-five percent of adolescent mothers in the intervention group were more likely to use contraception versus 22% in the comparison group (*p* ≤ 0.0001).	The school attendance rate amongst adolescent mothers in the intervention group was 77% versus 38% in the control group (*p* ≤ 0.0001).
Seitz and Apfel (1993)	Adolescent mothers who stayed at the Polly T McCabe Centre for more than 7 weeks had a lower repeat pregnancy rate at 24 months versus the adolescent mothers who stayed at the centre for 7 weeks only (12%, 6/50 vs. 36%, 19/52, *p* = 0.005).	-	Of the adolescent mothers who had not experienced a repeat pregnancy at 24 months, 69% completed high school compared to the 35% of the repeat adolescent mothers (*p* ≤ 0.005).
Solomon and Leifeld (1998)	At 24 months, 3 repeat pregnancies (10%) occurred in the intervention group in comparison to 11 (33%) in the control group (*p* = 0.006).	-	At 24 months, only 3 of 43 mothers in the intervention group dropped out of school in comparison to 12 of 29 in the control group (*p* = 0.002).
Steven-Simons et al. (1999)	At 24 months, the repeat pregnancy rate amongst implant users was 12% versus 46% in the control group (*p* ≤ 0.0001).	-	
Tocce et al. (2012)	At 6 months, there were no repeat pregnancies in the intervention group IPI in comparison to 9.9% in the control group. The IPI continuation rates at 6 and 12 months were 96.9% and 86.3%, respectively. At 12 months, the repeat pregnancy rate in the intervention group was 2.6% (4/153) in comparison to 18.6% (38/204) in the control group relative risk (5.0, 95% CI: 1.9–12.7). The repeat pregnancy at 12 months was predicted by not receiving IPI (OR 8.0, 95% CI: 2.8–23.0).	-	-

Note: Please see the full reference list of the article, Govender D, Naidoo S, Taylor M. Scoping review of risk factors of and interventions for adolescent repeat pregnancies: A public health perspective. Afr J Prm Health Care Fam Med. 2018;10(1), a1685. https://doi.org/10.4102/phcfm.v10i1.1685, for more information.

COCP, combined oral contraceptive pill; DMPA, depot medroxyprogesterone acetate; IPI, immediate postpartum implant; WCJF, Women’s Centre of Jamaica Foundation; LARC, long acting reversible contraception; OR, Odds ratio; CAMI, computer assisted motivational intervention.

Similarly, O’Sullivan and Jacobsen^34^ found that only 12% of the adolescent mothers who participated in the health care programme for first time adolescent mothers (behavioural skills development, contraceptive education, life option enhancement, sexuality, HIV and sexually transmitted infections (STI) education, infant care and education support attainment) had a repeat pregnancy in comparison to 28% of adolescent mothers in the control group at 18 months postpartum.

 A study carried out by Cox et al.^21^ in the USA also evaluated a hospital community-based medical home model for adolescent mothers and their children in which the outcomes of programme retention, health care utilisation, infant immunisation status, contraceptive use, repeat pregnancy, depressive symptoms, social support, school attendance and employment status were measured. The study design was a prospective single cohort study with the following programme components; infant care education, life orientation, social services support, family support groups, contraceptive education, day care and community outreach services. As there was no control group, benchmark comparison data were used through a systematic literature review. The adolescent repeat pregnancy rate was 14.7% at 12 months and 24.6% at 24 months. Cox et al.^21^ compared the findings of their study to those of Barnet et al.,^9^ who found that the repeat pregnancy rate amongst adolescent mothers in the intervention group was 45% at 24 months.

Numerous studies have focused on specific community-based health care centres for adolescent mothers with emphasis on secondary pregnancy prevention. For example, Solomon and Liefeld^25^ reported on the success of a family growth centre (FGC) where the repeat pregnancy rate amongst adolescent mothers in the intervention group was 12% in comparison to 41% in the control group in a 3-year longitudinal study. The FGC was designed using the Ecological Model and the Family Support Model, taking into consideration that adolescent pregnancy does not occur in a vacuum. Similarly, the Women’s Centre of Jamaica Foundation (WCJF) Programme for adolescent mothers found that the programme participants’ risk of a repeat pregnancy was reduced by 45% 4 years post evaluation.^35^ The WCJF programme components included community-based pregnancy prevention, parenting education, family planning services, life skills orientation, job training and placement, counselling services for adolescent fathers, and school support services.

By employing the ecological framework and social cognitive theory (SCT), Ford et al.^36^ evaluated a peer-centred prenatal programme for adolescent mothers. The study focused on birth weight, repeat pregnancy and educational outcomes at 12 months postpartum. The intervention included group therapy and prenatal education whilst the control group received the usual individual prenatal care. Whilst it can be argued that the repeat pregnancy differences between the intervention and control groups were not statistically significant, the repeat pregnancy rate in the peer-centred prenatal programme was lower than in the control group (13.4% vs. 15.9%)

Some authors argue that school-based programmes for adolescent mothers are also effective in reducing repeat pregnancies.^37,38^ For example, in a South Carolina high school, a school-based programme that provided comprehensive care found that the repeat pregnancy rate was 17% in the treatment group in comparison to 29% in the control group at 3 years after the previous birth.^37^ The school-based programme included medical services, social services, peer-based education, contraceptive education, parenting skills and educational attainment support for adolescent mothers. In a similar school-based programme, at the Polly T McCabe Centre, adolescent mothers who stayed for more than 7 weeks at the school-based centre had a lower repeat pregnancy rate at 24 months versus the adolescent mothers who stayed at the centre for 7 weeks only (12%, 6/50 vs. 36%, 19/52, *p* = 0.005).^38^ In addition, Corocan and Pillai’s^39^ meta-analysis of 16 studies focusing on adolescent repeat pregnancy prevention programs, which included school-based programs, revealed a 50% reduction in the likelihood of repeat pregnancies.

Apart from community and school-based interventions, home nurse visiting and motivational interviewing (MI) have also proven to be effective in delaying and reducing repeat pregnancies amongst adolescent mothers.^37,40,41^ The effects of a home-based mentoring programme on repeat pregnancy prevention for first time adolescent mothers was evaluated by Black et al.^41^ The mentorship was based on a ‘big sister approach’ and delivered over 19 home-based lessons. At 2 years postpartum, the adolescent mothers in the intervention group had a lower repeat pregnancy rate at 11% versus 24% in the control group. As with previous studies that have been discussed, the loss of participants at follow-up was significant because 18% of the mothers did not complete the 24-month evaluation.

Barnet et al.^42^ conducted a randomised trial in Baltimore, MD, USA, to evaluate the effectiveness of MI through a computer-assisted motivational intervention (CAMI) to prevent adolescent repeat pregnancies. Motivational Interviewing is a counselling approach that was used to help adolescent mothers find the motivation to facilitate positive behaviour change.^42^ The study recruited and trained counsellors on the computer-assisted MI. These counsellors provided motivation on use of contraception and condoms. The CAMI + group received education on infant development and care, feeding and nutrition, sexual and reproductive health, educational attainment and goal setting via biweekly home visits over a 24-month period. The CAMI-only group were visited at home on a quarterly basis. The CAMI and home visiting reduced the risk of rapid repeat adolescent pregnancies in the CAMI + group. In this respect, MI as an intervention can assist in reducing adolescent repeat pregnancies through behavioural change.^42^

According to Lewis et al.,^22^ contraception plays a vital role in the repeat adolescent pregnancy phenomenon. The frequency of research studies on contraceptive implants and repeat pregnancy rates in adolescents has continued to increase.^4,22,26,43,44^ Some of the findings that were most compelling arose from a study by Tocce et al.,^3^ which revealed that only 2.6% of adolescent mothers (*n* = 4/153; relative risk = 5.5; 95%; CI: 1.9 – 12.7) with immediate postpartum etonogestrel implant insertion experienced a repeat pregnancy at 12 months versus 18.6% (38/204) of the control participants. A retrospective study in the USA in the Medstar Washington Hospital by Damle et al.^43^ found reduced repeat pregnancy rates at 24 months amongst adolescent mothers who had initiated a long-acting reversible contraceptive within 8 weeks of delivery. A recent study by Cohen et al.^44^ also revealed that the repeat pregnancy rate is lower amongst adolescent mothers who initiate postpartum long-acting reversible contraceptives (LARC). In this regard, contraceptive implants could be an important intervention for adolescent repeat pregnancy.

## Discussion

Adolescent repeat pregnancy has been studied extensively in other countries but in the African continent, studies on this phenomenon are scarce. With regard to the risk factors for adolescent repeat pregnancy, the ecological framework best explains this phenomenon. The results obtained by Mphatswe et al.^8^ on the prevalence and the risk factors on adolescent repeat pregnancy in KwaZulu-Natal, South Africa, were similar to that of other published studies around the globe. The scoping review of the literature demonstrates that interventions on secondary pregnancy prevention have positive outcomes for adolescents. These comprehensive interventions offered a wide range of medical services, education and psychosocial services which targeted repeat pregnancy, school enrolment and contraceptive uptake. Furthermore, the literature indicates that the ecological framework provides guidance on strategies to prevent adolescent repeat pregnancy.

The vast majority of interventions in this scoping review recruited participants from clinics, hospitals and community centres. In this regard, adolescent mothers not seeking health care would have been excluded. The findings of this scoping review illustrate the need for adolescent repeat pregnancy interventions to shift from individuals to the wider social and community context that influences adolescent sexual and reproductive health.

The FGC intervention by Solomon and Leifeld^25^ sought to change adolescent behaviour with emphasis on socio-ecological factors. The theoretical approaches used in the design of the FCG included the Ecological Model and the Family Support Model. The macroscopic ecological factors included social support, parenting skills and life stresses, whilst microscopic factors included home stimulation and child’s temperament. Activities within the Ecological and Family Support Models included social and recreational, grandmother support groups, parenting classes, transportation services and developmental day camps.^24^

Interventions by Tocce et al.,^4^ Lewis et al.^22^ and Steven-Simons et al.^26^ drew attention to the use of contraception in adolescents. In interpreting the results, it is noteworthy that contraception as an intervention plays a vital role in the adolescent repeat pregnancy phenomenon. In this regard, healthcare providers need to support adolescents in the access and use of contraceptives. The reported levels of contraception uptake and use are low amongst both married and unmarried adolescents.^45,46^ This implies that, though most married adolescents do not want a pregnancy, their contraceptive use is lower than that of sexually active unmarried adolescents.^45^

In a nutshell, there is an unmet need for contraception amongst adolescents in general. For example, a study in Nigeria by Ahanonu^47^ found that health care providers did not approve of adolescent premarital sex and that they, therefore, felt that prescribing contraceptives to adolescents was promoting promiscuity. Other factors that influence contraception use include: (1) sexual experiences, (2) psychosocial development, (3) gender issues, (4) previous health care experiences, (5) access to health care services, (6) access to information, (7) health and education structures and (8) social and cultural norms.^46^ In the South African context, Ehlers’s^48^ explorative descriptive survey study found that only 48% of 250 adolescent mothers surveyed had used contraception.

A ‘single one-size-fits-all’ intervention for adolescent repeat pregnancy prevention is unlikely as different strategies were employed by the intervention programmes in this scoping review. In short, health care services need to be tailored to the needs of pregnant and parenting adolescents. Interventions can be implemented at various sites, including clinics, schools and community centres.

## Study limitations

For practical reasons, only articles written in English were considered for this scoping review. The authors acknowledge that important published research may have been omitted using the method outlined in the search methodology. Rather, the review was designed to further illuminate the phenomenon of adolescent repeat pregnancy and to provide an understanding of what is known about the risk factors of and the interventions for adolescent repeat pregnancy.

## Conclusion and recommendations

Health services, health care workers and health information are essential building blocks of the public health system. According to the World Health Organization (WHO),^49^ the ‘…strengthening of health systems is everybody’s business’. In this regard, the improvement of health care services for adolescent mothers is indeed everybody’s business. Based on this review, it can be concluded that health care providers play an important role in the prevention of adolescent repeat pregnancies as it was shown that, across various studies, adolescent mothers who received medical, psychosocial, educational and family planning support experienced lower rates of repeat pregnancies.

To address the shortage of literature on adolescent repeat pregnancy in South Africa, future research needs to focus on this phenomenon and the strengthening of comprehensive health care services for adolescent mothers. Recommended for inclusion in future interventions, the Pinzon and Jones^50^ paediatric policy statement for the care of adolescent parents and their children highlights the following points: (1) continuity of care (creation of a medical home model), (2) provision of a multidisciplinary approach to care (nursing, social services, nutritional care, developmental screening services), (3) breastfeeding support, (4) contraception, (5) youth development, (6) promoting academic achievement, (7) promoting a healthy lifestyle, (8) addressing mental health issues, (9) parenting skills, (10) development of support groups and (11) active involvement of adolescent fathers. Adolescent repeat pregnancy is a huge burden on the adolescent mother, her family, her community and her country of residence.^51^ According to Morris and Rushwan,^46^ health care workers need to ‘move away from being part of the problem to being part of the solution’ (p. S41).
